# Supplementary material for: An open-source framework for large-scale, flexible evaluation of biomedical text mining systems
Source: J Biomed Discov Collab. 2008 Jan 29;3:1. doi: 10.1186/1747-5333-3-1 (PMC2276192; doi:10.1186/1747-5333-3-1)
Supplement: Additional file 1 — Evaluation platform source code. The additional file contains the software evaluation framework discussed in this paper. The most current release can be downloaded from SourceForge.net [1]. See the accompanying README file for instructions on installation and use. Also included in the distribution is a collection of UIMA wrappers for some commonly used BioNLP tools and annotated corpora. [file 1747-5333-3-1-S1.zip › bionlp_uima_repository-1.0.1/docs/api/src-html/edu/uchsc/ccp/util/nlp/annotation/comparison/IgnoreSpanComparator.html]

```
001    package edu.uchsc.ccp.util.nlp.annotation.comparison;
002    
003    import java.util.List;
004    
005    import edu.uchsc.ccp.util.nlp.annotation.Span;
006    
007    /**
008     * 
009     * Use this span comparator when you do not care to compare spans. For example, this may be of use when doing document-level comparisons.
010     * 
011     * @author williamb
012     * 
013     */
014    public class IgnoreSpanComparator extends SpanComparator {
015    
016        /**
017         * Ignores the spans and returns 0 always.
018         */
019        @Override
020        public int compare(Span span1, Span span2) {
021            return 0;
022        }
023    
024        /**
025         * Ignores the span lists and returns 0 always.
026         * @param spanList1
027         * @param spanList2
028         * @return
029         */
030        @Override
031        public int compare(List<Span> spanList1, List<Span> spanList2) {
032            return 0;
033        }
034    
035    }
```
